# Mental health index of the elderly population in Medellín (Colombia)−2021: a factorial analysis

**DOI:** 10.3389/fpsyg.2024.1336562

**Published:** 2024-06-26

**Authors:** Sandra Patricia Ochoa-Nieto, Luisa María Benjumea-Gómez, Doris Cardona-Arango, Ángela Segura-Cardona, Alejandra Segura-Cardona, Carlos Robledo-Marín

**Affiliations:** ^1^Department of Public Health, CES University, Medellín, Colombia; ^2^Department of Epidemiology, CES University, Medellín, Colombia; ^3^Department of Demography, Public Health Observatory Research Group, CES University, Medellín, Colombia; ^4^Department of Epidemiology, Research Group in Epidemiology and Biostatistics of the CES University, Graduate School of CES University, Medellín, Colombia; ^5^Department of Epidemiology and Biostatistics, CES Clinical Psychology Service, CES University, Medellín, Colombia; ^6^Department of Humanities, Fundación Opción Colombia (FUNDACOL), CES University, Medellín, Colombia

**Keywords:** mental health, hopelessness, resilient coping, self-esteem, self-compassion, quality of life, depression

## Abstract

**Introduction:**

There are several risk factors associated with developing mental disorders among people over 60 years of age. Mental health encompasses multiple domains or capacities, which can comprise the psychological wellbeing of older people. Therefore, resilient coping, self-compassion, self-esteem, hopelessness, quality of life, and depression are considered the characteristics and adaptive mechanisms that bring together the main risk and protective factors for imbalance in mental wellbeing.

**Objective:**

This study aimed to establish the demographic, social, and family factors, as well as the clinical characteristics and lifestyle habits that influence the mental health of the elderly people in the city of Medellín, in the year 2021, to build a mental health index.

**Methodology:**

This study adopts a quantitative approach, employs retrospective temporality, and utilizes secondary sources. A cross-sectional survey was carried out with the SABAM Project (health and mental wellbeing of older adults from five cities in Colombia in 2021 by its Spanish acronym) database (secondary source), which is part of the research group “Public Health Observatory” of CES University (Medellín, Colombia). The database comprised 500 records of people aged over 60 years from the city of Medellín in 2021. While constructing the index, a principal component analysis was used, along with the Varimax method for factor analysis rotation.

**Results:**

The median age of the general population was 67 years (IQR 63–72); for men, the median age was 66 years (IQR 63–71) and for women, the median age was 67 years (IQR 63–72). An association was found between low socioeconomic levels, alcohol consumption, and the level of higher education (university—postgraduate) with low levels of the Mental Health Index in people over 60 years of age in Medellín during the year 2021.

**Conclusion:**

A quantitative model was developed to predict either a positive or negative prognosis in the mental wellbeing of the population over 60 years of age in the city of Medellín. The model was also used for the development of a sociodemographic profile highlighting the impact on mental health among people over 60 years of age with limited economic resources.

## 1 Introduction

The WHO has proposed the following meaning of Mental Health: “…a dynamic state that is expressed in everyday life through behavior and interaction, allowing the subject to deploy their emotional, cognitive and mental resources to navigate, work, and establish meaningful relationships with an echo in the community” (World Health Organization, 2004). In this regard, mental health is not only considered the absence of psychological disease but also includes wellbeing, the effective functioning of individuals, and their interaction with the community (Carrazana, 2003).

Nussbaum in her book “Creating Capabilities” considers that the person has essential core capabilities for their integral development: life, physical health, physical integrity, senses, imagination and thoughts, emotions, practical reasons, affiliations, relationship with other species, play, control over one's environment through political and material (Nussbaum, 2012). These abilities are associated with individual humans and are based on the construction of quality of life, defined by the WHO as the perception that an individual has of their position in life in the context of culture and value systems in which they live and about their goals, expectations, standards, and concerns (WHOQOL Group, 1995). With these two concepts, it could be inferred that mental health is a construct of human capabilities that allow the development of a quality of life adapted to the needs of everyone.

In this context, it could be considered that the absence of any of the capabilities would produce an imbalance in the quality of life, which would predispose individuals to experiencing some psychological, biological, and/or social discomforts, thereby creating a conducive environment for the appearance of mental illness (Sánchez Díaz, 2005). Human aging constitutes a multidimensional, heterogeneous process inherent to human beings and is irreversible, which constitutes a complex and individual process in which the person undergoes biological and psychological changes that have repercussions on social, economic, and cultural lifestyle during their life (Alvarado García and Salazar Maya, 2014). It is a diverse process that depends on an individual experience faced in diverse ways according to social, cultural, and personal contexts but has one factor in common: a state of deterioration in several vital aspects (Dulcey-Ruiz, 2010). However, health levels are not aligned with increased longevity, and the latest global report on aging (World Health Organization, 2015) and health highlights large inequities and difficulties in health among older adults, particularly in mental health (Officer et al., 2016). More than 20% of people over 60 years suffer from some mental or neural disorder, with dementia and depression being the most common (Sánchez Díaz, 2005). In addition, 6.6% of disability in this group are attributed to mental disorders and nervous system disorders, representing 17.4% of years lived with disability in the elderly population (World Health Organization, 2017).

There are several risk factors associated with developing mental disorders among the older adult population; these include social isolation, physical or psychological abuse, associated pathologies, genetic predisposition, stressful situations, and a lack of physical exercise, among others (Palloni and McEniry, 2007). On the other hand, some protective factors for mental health in older people are functionality in daily activities, active social relationships, spirituality, male sex, and resilience capacity (Chavarro-Carvajal et al., 2017). This suggests that there are multiple domains of mental health, also considered as capabilities, that can encompass the mental health of older people. It is believed that resilient coping, self-compassion, self-esteem, hopelessness, quality of life, and depression are characteristics and adaptive mechanisms that gather the main risk factors and protectors for imbalance in mental wellbeing.

Initially, resilience is defined as the ability to recover from adversity and successfully adapt to stressful situations (Resnick, 2014). Distinct types of resilience are described, such as general health (Sanders et al., 2008), psychological (Boardman et al., 2008), emotional (Chow et al., 2007), and dispositional (Rossi et al., 2007). The Brief Resilience Coping Scale (BRCS) is taken as the measurement instrument for measuring this domain. It is defined as a 4-item measure designed to capture tendencies to cope with stress in a highly adaptive way. The BRCS is a short unidimensional scale that aims to assess people's ability to cope with stress adaptively and is easy to apply and interpret (Tomas et al., 2021).

Self-compassion can be defined as a compassionate attitude toward oneself in the face of suffering (Simón, 2017). Neff (2003) has highlighted that self-compassion involves warmth and understanding toward oneself, rather than passing judgment when one is suffering, noting that compassion has interrelation components that can manifest through pain and suffering. He also highlights the three main components of self-compassion: kindness to oneself, which involves self-affirmation, happiness, and affection, contrasted with self-criticism, which involves a feeling of hostility, and constant self-demand (Chow et al., 2007). Shared humanity implies recognition of one's own and general elements with humanity (Chow et al., 2007). The concept of mindfulness allows us to understand that one's being is not separate from what surrounds it, but rather exists by various elements that constitute it (Chow et al., 2007). Therefore, it is evident that self-compassion is different from self-complacency. While self-compassion allows one to recognize human error, one's error without the need to adopt defensive attitudes, self-complacency implies reactivity, such as indifference or resignation, resulting in stagnation and avoidance of responsibility. In contrast, self-compassion presents a fair observation of what happens at the precise moment, with an active interest in the common good (Strauss et al., 2016). Self-compassion is evaluated using the “Self-Compassion Scale.” The original version contains 26 items, which measure six components of self-compassion: kindness to oneself, self-judgment, humanity, isolation, mindfulness, and overidentification (Garcia-Campayo et al., 2014).

Self-esteem is understood as the attitude and posture toward oneself; however, when defining it as an attitude, anthropological and psychological assumptions are accepted. Similarly, self-esteem invites us to study unfamiliar cultural aspects and personality traits that help build it (Naranjo Pereira, 2011). Currently, several instruments measure self-esteem but one of the most commonly used is Rosenberg's Self-Esteem Scale, which has been validated and translated into different languages. Rosenberg understands self-esteem as a feeling toward oneself that can be either positive or negative and constructed by personal and social characteristics. Initially, the scale was directed only at adolescents but today it is used in different age groups. It is quick to apply and has 10 items divided into positive or negative ratings (Chen et al., 2004).

Hopelessness is denoted as the tendency to make negative inferences about the causes, consequences, and implications of a person and their inferences about their negative life events. Hopelessness has been considered a crucial factor in determining vulnerability, types of depression, adjustment disorders, and contemplating the risk of suicidal thoughts (Calvete et al., 2007). Thus, hopelessness somewhat predicts negative consequences and it could be concluded that if an event goes wrong it can cause a failure in the person as such, being able to make a fatalistic interpretation of problems, affecting different areas of life, creating an environment of guilt and the illusion that this situation will continue happening in the future (Hankin and Abramson, 2001). The Beck Hopelessness Scale was used as an instrument in which pessimism and negative attitudes toward the future are detected in patients with depression and risk of suicide, and it also evaluates difficulties in achieving success in life. This scale is modeled under three factors, namely, affective, motivational, and cognitive factors, that are evaluated (Beck et al., 1974).

Depression is not a normal part of aging and should be considered a serious and disabling medical disorder. Depression is a common disorder in older adults that is associated with an increase in disability and costs, with negative health outcomes over time. Antidepressant treatments in the form of medication or psychotherapy are available, but a considerable proportion of those treated do not respond completely, and relapse or recurrence of symptoms is frequent among those who recover. Therefore, successful prevention would avoid these negative outcomes (Almeida, 2014). It is considered that the depressive disorder with the highest recurrence is major depression. It is defined in DSM-5 based on the presence of five or more core depressive symptoms over a 2-week period, which includes depressed mood or loss of interest or pleasure, along with significant weight loss or gain (without dieting) or change in appetite, insomnia or hypersomnia, agitation or psychomotor retardation, fatigue or loss of energy, feelings of worthlessness or inappropriate guilt, decreased ability to think or concentrate or indecision, and recurrent thoughts of death or suicide (American Psychiatric Association, 2014). Major depression, minor depressive symptomatology, and dysthymia are the two disorders that follow in frequency (Devita et al., 2022). “Minor depression” is sometimes known as “subsyndromal or subthreshold depression.” It is not a designated diagnostic category in DSM-5 but is denoted as a section under the category “other specified depressive disorders”. Dysthymia (alternatively known as persistent depressive disorder in DSM-5) is a chronic form of depression that is less severe than major depression and lasts 2 or more years (Casey, 2017).

Finally, quality of life is defined by WHO as follows: “the perception that a person has about their position in life within the cultural context and the value system in which they live and concerning their goals, expectations, standards, and concerns. It is a broad concept that is complexly traversed by the person's physical health, their physiological state, the level of independence, their social relationships, and the relationship they have with their environment” (World Health Organization, 2020). It is evident that it is an ambiguous and subjective construct. Thus, Lawton considers quality of life as “the multidimensional evaluation, both by intrapersonal and socio-normative criteria, of an individual's person-environment system in past, present, and anticipated time” (Lawton, 1991), making evident the need for standards that allow measurement seeking objective follow-up (Fumincelli et al., 2019). In this regard, there are three main models to measure the quality of life: (1). DIMENSIONS: a. objective, or based on external observations of the individual. b. subjective, or based on the individual's psychological responses. (2). DOMAINS: a. physical health, general or specific to each disease. b. psychological: mental wellbeing. c. social or interpersonal relationships and community. (3). INSTRUMENTS: a. generic, or common to any population. b. idiopathic or for specific populations (Netuveli and Blane, 2008). In this way, with the design of instruments that allow the measurement of quality of life criteria, the World Health Organization developed the Quality of Life Group (World Health Organization Quality of Life Assessment - WHOQOL) in 1996, allowing the design of an instrument that integrates all the previously mentioned elements, thus facilitating the evaluation of the quality of life that can serve as a measure of results in research related to the comparative benefits of different therapeutic methods (Madero-Cabib et al., 2022). Taking all this into account, Cardona concludes “The concept of quality of life has gone from being an abstract form of wellbeing and happiness to being considered operationally as the standard of living and living conditions, according to different disciplines and areas of knowledge, both philosophical and ethical, as well as economic, social, and cultural” (Cardona and Agudelo, 2009).

This study sought, using the measurement of the six domains described, to construct a Mental Health Index that contributes to the early detection of positive or negative risk factors, as well as associated sociodemographic and family factors, adapted to older people in the city of Medellín in 2021.

## 2 Methodology

A quantitative cross-sectional study with retrospective temporality was developed using secondary information from the SABAM Project (health and mental wellbeing of the elderly person from five cities in Colombia in 2021 by its Spanish acronym) database of the Public Health Observatory research group of the CES University. The SABAM project was developed in 2021 to compare mental health (suicidal behavior, psychoactive substance use, and gambling addiction) and mental wellbeing (psychological wellbeing, happiness, and quality of life) among older adults residing in five cities of Colombia (Bucaramanga, Medellín, Pereira, Popayan, and Santa Marta), with the goal of achieving active aging, included in the main SABAM study in 2021, grouped into several components in the data collection instrument to include the dependent, independent, and confounding variables (Supplementary material).

The reference population was 408,879 older adults over 60 years of age residing in the city of Medellín according to the population census projection of DANE (National Administrative Department of Statistics by its Spanish acronym) for the year 2018–2070. The sample, comprising 500 individuals, was obtained from a secondary source and is made up of all the records in the database. It is important to clarify that the SABAM Project obtained its information from five cities in Colombia, where they conducted two-stage analyses and stratified cluster sampling. In the city of Medellín, it is distributed by communes and neighborhoods in the urban area with a sample calculation of 500 surveys, where a finite population formula was implemented with a confidence level of 95%, a significance of 5%, and a confidence coefficient of 1.96.

The variables in this study were divided into four principal areas (for detailed information about the variables in each group, see Supplementary material). The first one is constituted by sociodemographic and family variables that compose those personal characteristics of age, sex, health regime, place of residence, occupation, income, level of education, and insurability to SGSSS (Colombian General System of Social Security in Health by its acronym in Spanish). The second group includes clinical variables and lifestyle habits. Physical illness, global functionality, and some lifestyle habits such as alcohol and psychoactive substance consumption are included. The third group includes interpersonal relationship variables such as the absence of a partner, family functionality, and deficient social support. The fourth area is composed of mental health variables, which are constituted by six spheres of emotional wellbeing: (a) depression, measured with the Center for Epidemiological Studies Depression Scale—CES D, (b) quality of life, analyzed from the WHOQOL-OLD Scale, (c) hopelessness, which is obtained from the measurement of Beck's Hopelessness Scale, (d) resilient coping, abstracted from the responses to the Brief Resilience Coping Scale, (e) self-esteem with data obtained from Rosenberg's Self-Esteem Scale, and finally (f) self-compassion was extracted from the items on the Self-Compassion Scale (SCS).

The statistical software used for database analysis was IBM SPSS Statistics Version 26 and Jamovi 2.3.18. The data analysis was divided into four stages. For the description of social and family characteristics of the population of older people in the city of Medellín in 2021, sociodemographic and family description variables were selected from the database, applying a univariate analysis for frequency measures and descriptives. Subsequently, a mental health index is constructed through a factorial analysis using the principal components method, finally obtaining the dependent variable named Mental Health Index in person over 60 years in Medellín (ISMPM60M). Next, the statistical association between sociodemographic and family variables with ISMPM60M was calculated through bivariate analysis obtaining crude prevalence ratios (PRc) for each association and confirmation with chi-square statistical tests with confidence intervals (CI) at 95%. In multivariate analysis, associations with statistical significance observed in bivariate analysis (*p* < 0.025) were examined, with logistic regression used to obtain adjusted prevalence ratios (PRa).

## 3 Results

Participants in this study presented a sex distribution of 60.2% of female participants and 39.8% of male participants in a 1:1.5 ratio. The median age of the population was found to be 67 years, for men, the median age was 68 years, and for women, the median age was 66 years. Age distribution ranges for ~60% of men were found to be between 60 and 69 years, followed by 36% of men between 70 and 79 years, and finally, those over 80 years comprised the remaining percentage (Table 1). A similar distribution among women was found with the majority of 70% of women between the age range of 60–69 years, 22% of women between 70 and 79 years, and the remaining 8% of women over 80 years (Table 2).

**Table 1:** Percentage distribution of the elderly population, according to demographic and social characteristics, by sex in Medellín, 2021.

**Variable**	**Men**		**Women**		**Total**	
	**#**	**%**	**#**	**%**	**#**	**%**
**Age** ^*^	68 (64–73)		66 (63–71)		67 (63–72)	
60–69	116	58.3%	212	70.4%	328	65.6%
70–79	72	36.2%	65	21.6%	137	27.4%
80–89	6	3.0%	21	7.0%	27	5.4%
90–99	4	2.0%	3	1.0%	7	1.4%
100 and more	1	0.5%	0	0.0%	1	0.2%
**Marital status**						
Single	46	23.1%	64	21.3%	110	22.0%
Married—union	84	42.2%	131	43.5%	215	43.0%
Separated—divorced	39	19.6%	41	13.6%	80	16.0%
Widowed	30	15.1%	65	21.6%	95	19.0%
**Education**						
Primary school	96	48.2%	165	54.8%	261	52.2%
High school	49	24.6%	67	22.3%	116	23.2%
Tech—college	17	8.5%	22	7.3%	39	7.8%
Postgraduate	23	11.6%	18	6.0%	41	8.2%
None	14	7.0%	29	9.6%	43	8.6%
**Economic income**						
< 1 SMMLV^**^	93	46.7%	106	35.2%	199	39.8%
Between 1 and 2 SMMLV	11	5.5%	5	1.7%	16	3.2%
More than 2 SMMLV	2	1.0%	0	0.0%	2	0.4%
None	93	46.7%	93	63.1%	283	56.6%
**Socioeconomic level**						
Low (1–2)	121	60.8%	211	70.1%	332	66.4%
Medium (3–4)	78	39.2%	90	29.9%	168	33.6%
**SGSSS** ^***^						
**Health program**						
Contributory	79	39.7%	111	36.9%	190	38.0%
Subsidiary	115	57.8%	186	61.8%	301	60.2%
Uninsured	5	2.5%	4	1.3%	9	1.8%
**Retirement program**						
Affiliate	76	38.2%	75	24.9%	151	30.2%
Non-affiliate	123	61.8%	226	75.1%	349	69.8%

^*^Median and Interquartile Range (IQR) values.

^**^Colombian Current Legal Monthly Minimum Salary by its acronym in Spanish.

^***^Colombian General System of Social Security in Health by its acronym in Spanish.

**Table 2:** Percentage distribution of the elderly population, according to interpersonal and clinical relationship characteristics, by sex in Medellín, 2021.

**Variable**	**Men**		**Women**		**Total**	
	**#**	**%**	**#**	**%**	**#**	**%**
**Absent partner**						
Yes	55	27.64%	121	40.20%	176	35.20%
No	144	72.36%	180	59.80%	324	64.80%
**Deficient social support**						
Yes	11	5.53%	10	3.32%	21	4.20%
No	188	94.47%	291	96.68%	479	95.80%
**Familiar APGAR**						
Functional	127	63.82%	217	72.09%	344	68.80%
Low dysfunction	32	16.08%	58	19.27%	90	18.00%
Moderate dysfunction	15	7.54%	6	1.99%	21	4.20%
High dysfunction	25	12.56%	20	6.64%	45	9.00%
**Functionality—BARTHEL scale**						
Dependence	0	0.00%	1	0.33%	1	0.20%
Low dependence	36	18.09%	33	10.96%	69	13.80%
Moderate dependence	0	0.00%	1	0.33%	1	0.20%
High dependence	1	0.50%	0	0.00%	1	0.20%
Independent	162	81.41%	266	88.37%	428	85.60%
**Physical illness**						
Yes	26	13.07%	60	19.93%	86	17.20%
No	173	86.93%	241	80.07%	414	82.80%
**Alcohol abuse**						
Yes	14	7.04%	40	13.29%	54	10.80%
No	185	92.96%	261	86.71%	446	89.20%
**Consumption of psychoactive substances**						
Yes	68	34.17%	68	22.59%	136	27.20%
No	131	65.83%	233	77.41%	364	72.80%

The present Mental Health Index was constructed using the information on the mental health status of the studied population and from the data collected, and 80% of the records from the database of the studied population were taken and selected by simple random sampling.

**Figure 1:**
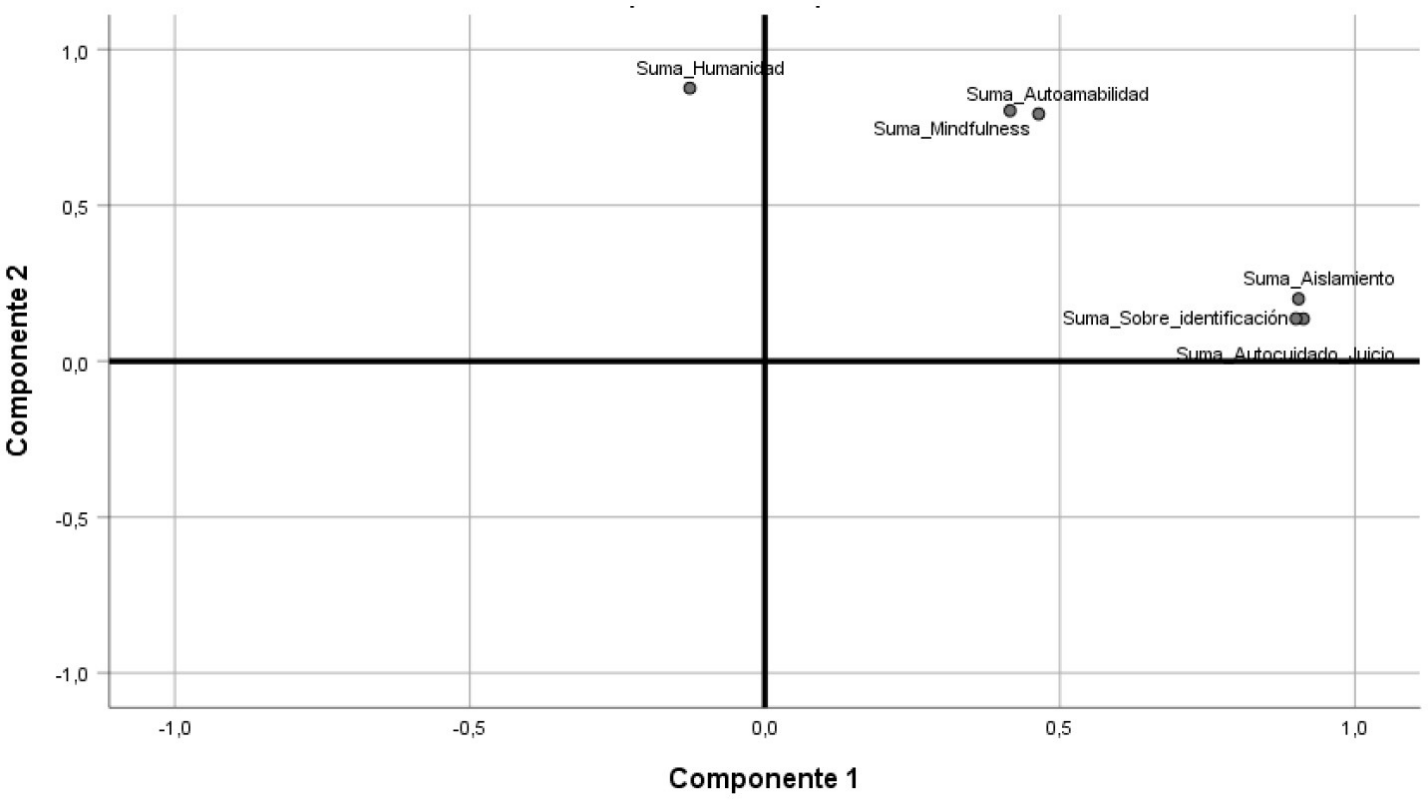
Components in rotated space for the mental health index of people over 60 years of age in the city of Medellín, 2021.

Once the principal component analysis (PCA) was performed for the mental health variables, a factorial structure was obtained with a result of two components, each with three variables, the first one called individual component and the second one called existential component, which explains 79.6% of the total variance. For this item, variables related to self-judgment, isolation, overidentification, self-kindness, humanity, and mindfulness were considered, with this last variable as the ability to live in the present moment within the studied population (see Figure 1).

For content validity, it was performed with the varimax rotation method with Kaiser normalization, and sample adequacy through the KMO and Bartlett index, where the result was 0.796, confirming that the sample is adequate for factorial analysis. On the other hand, Bartlett's sphericity test allowed for the rejection of the null hypothesis that states that variables are not related in the studied population with a chi-square of 2,070.105 and a *p*-value of 0.000.

The result can be grouped into six variables as follows: the individual component contains self-judgment, isolation, and overidentification. The existential component includes self-kindness, humanity, and mindfulness (see Table 3).

**Table 3:** The main components of the mental health index in people over 60 years of age in Medellín, 2021.

	**Coping mechanism**	
**Variable**	**Individual (component 1)**	**Existential (component 2)**
Self-judgment	0.913	
Isolation	0.904	
Overidentification	0.900	
Self-kindness		0.793
Humanity		0.876
Mindfulness		0.804

Once the main components are identified, each component is established as a variable for calculating the Mental Health Index in people over 60 years in Medellín (MHI60YM) in the year 2021. The index definition is the value obtained from summing up components with values in a range from −5 to 5. With the data obtained, we can establish two categories separated by value zero from the range: >0 is considered a Positive Mental Health Index and < 0 is a Negative Mental Health Index.

Given that by theoretical definition, the characteristics comprising each component are coping mechanisms in mental health, they can be grouped as individual and existential mechanisms. Therefore, it is considered that obtaining a sum of these capabilities defines a state of mental wellbeing that allows individuals to cope with everyday conditions. This results in the establishment of two states: positive mental health state (with values above zero) and negative mental health state (values below zero).

The Mental Health Index, which is defined as a new variable, showed a general distribution, with 55.2% of the total sample exhibiting negative MHI60YM and 44.8% exhibiting positive MHI60YM.

Prevalence ratios (PR) indicate that demographic and socioeconomic factors associated with the Mental Health Index for people over 60 years in Medellín in *r* 2021 include variables comprising the marital status, level of education, socioeconomic stratum, and SGSSS regime. All the above factors show statistically significant values (*p* < 0.025).

As shown in Table 4, the clinical factors and lifestyle habits (Table 5) associated with the Mental Health Index for people over 60 years in Medellín for the year 2021 are the variables that include the absence of a partner, family APGAR, alcohol abuse, and consumption of psychoactive substances. All the above with statistically significant values (*p* < 0.025).

**Table 4:** Absolute and percentage distribution of the mental health index of people over 60 years of age according to demographic and socioeconomic characteristics in Medellín, 2021.

**Factor**	**MHI60YM**				**PRc**	**CI (95%)**		***X^2^***	***p***
	**Positive**		**Negative**			**L**_l_	**L**_u_		
	**No**.	**%**	**No**.	**%**					
**Sex**									
Male	100	44.84	98	35.51	0.670	0.467	0.962	4.720	0.030
Female	123	55.16	178	64.49	–	–	–		
**Age**									
Average (SD)^*^	68.8	6.90	67.7	6.68	0.950	0.950	1.001	3.524	0.60
Median (IQR)^*^	68	63.5–73	66	63–71					
**Marital status**									
Married—union	94	42.15	121	43.84	1.064	0.655	1.730	15.9	0.001^*^
Separated—divorced	23	10.31	57	20.65	2.049	1.091	3.850		
Single	63	28.25	46	16.67	0.594	0.342	1.034		
Widowed	43	19.28	52	18.84	–	**–**	**–**		
**Education**									
Primary school	114	51.12	146	52.90	0.686	0.350	1.345	28.523	< 0.001^*^
High school	64	28.70	52	18.84	0.429	0.207	0.885		
Tech—college	26	11.66	13	4.71	0.268	0.107	0.669		
Postgraduate	4	1.79	37	13.41	4.955	1.482	16.572		
None	15	6.73	28	10.14	**-**	**-**	**-**		
**Socioeconomic level**									
Low (1–2)	102	45.54	66	24	2.660	1.816	3.896	25.249	< 0.001^*^
Medium (3–4)	122	54.46	210	76	**–**	**–**	**–**		
**Social security in health**									
Contributive	99	44.39	90	32.61	0.257	0.052	1.270	8.578	0.014^*^
Government subsidized	122	54.71	179	64.86	0.419	0.086	2.052		
Uninsured	2	0.90	7	2.54	**–**	**–**	**–**		
**Retirement program**									
Yes	56	25.11	94	34.06	1.513	1.024	2.236	4.326	0.038
No	167	74.89	182	65.94	**–**	**–**	**–**		

MHI60YM, Mental Health Index in people over 60 years of age in Medellín; PRc, prevalence ratio—crude; X^2^, chi-squared; CI, confidence interval; Ll, lower limit; Lu, upper limit.

^*^p statistically significant (*p* < 0.025).

**Table 5:** Absolute and percentage distribution of the Mental Health Index of people over 60 years of age according to clinical characteristics and lifestyle habits in Medellín, 2021.

**Factor**	**MHI60YM**				**PRc**	**CI (95%)**		***X^2^***	***p***
	**Positive**		**Negative**			**L**_l_	**L**_u_		
	**No**.	**%**	**No**.	**%**					
**Physical illness**									
Yes	43	19.28	42	15.22	0.734	0.461	1.169	1.44	0.193
No	180	80.72	234	84.78	–	–	–		
**Barthel scale**									
Independent	199	89.24	229	82.97	0.612	0.364	1.031	3.409	0.065
Dependent	24	10.76	47	17.03	–	–	–		
**Absent partner**									
Yes	97	43.50	78	28.26	0.506	0.349	0.735	12.850	< 0.001^*^
No	126	56.50	198	71.74	–	–	–		
**Familiar APGAR scale**									
Functional	170	76.23	173	62.68	1.058	0.568	1.969	17.806	< 0.001^*^
Low dysfunction	26	11.66	64	23.19	2.573	1.226	5.400		
Moderate dysfunction	4	1.79	17	6.16	4.443	1.291	15.294		
High dysfunction	23	10.31	22	7.97	–	–	–		
**Deficient social support**									
Yes	11	4.93	9	3.26	0.596	0.246	1.440	1.324	0.250
No	212	95.07	267	96.74	–	–	–		
**Alcohol abuse**									
Yes	9	4.04	45	16.30	4.654	2.222	9.748	16.614	< 0.001^*^
No	214	95.96	231	83.70	–	–	–		
**Psychoactive substances consumption**									
Yes	79	35.43	56	20.29	0.458	0.307	0.684	14.557	< 0.001^*^
No	144	64.57	220	79.71	–	–	–		

MHI60YM, Mental Health Index in people over 60 years of age in Medellín; PRc, prevalence ratio—crude; X^2^, chi-squared; CI, confidence interval; Ll, lower limit; Lu, upper limit.

^*^p statistically significant (*p* < 0.025).

When adjusting the measures of the factors associated with the Mental Health Index and calculating the adjusted PRs for the variables with statistical significance resulting from bivariate analysis, it is evident how in the adjusted PRs, the variables of marital status, absence of a partner, SGSSS regime, and consumption of psychoactive substances show insignificant association strength given by *p*-values > 0.025 (Table 6).

**Table 6:** Prevalence ratio of the Mental Health Index in people over 60 years of age in the city of Medellín (2021) according to the associated factors.

**Factor**	**PRc**	**CI 95%**		**PRa**	**CI 95%**		**X^2^**	***p***
		**L**_l_	**L**_u_		**L**_l_	**L**_u_		
**Marital status**								
Married—union	1.064	0.655	1.73	0.987	0.539	1.804	3.863	0.277
Separated—divorce	2.049	1.091	3.85	1.643	0.802	3.366		
Single	0.594	0.342	1.034	0.838	0.447	1.572		
Widowed	1.000	–	–	1.000	–	–		
**Education**								
Primary school	0.686	0.35	1.345	0.998	0.476	2.092	14.450	0.006^*^
High school	0.429	0.207	0.885	0.757	0.337	1.699		
Tech or college	0.268	0.107	0.669	0.463	0.165	1.301		
Postgraduate	4.955	1.482	16.572	5.309	1.506	18.711		
None	1.000	–	–	1.000	–	–		
**Socioeconomic level**								
Low (1–2)	2.66	1.816	3.896	2.003	1.292	3.107	9.631	0.002^*^
Medium (3–4)	1.000	–	–	1.000	–	–		
**Social security in health**								
Contributive	0.257	0.052	1.27	0.353	0.066	1.896	1.481	0.477
Subsidiary	0.419	0.086	2.052	0.361	0.068	1.921		
Uninsured	1.000	–	–	1.000	–	–		
**Absent partner**								
Yes	0.506	0.349	0.735	0.789	0.467	1.333	0.786	0.375
No	1	–	–	1.000	–	–		
**Familiar APGAR scale**								
Functional	1.058	0.568	1.969	0.726	0.358	1.471	13.076	0.004^*^
Low dysfunction	2.573	1.226	5.4	1.795	0.797	4.045		
Moderate dysfunction	4.443	1.291	15.294	2.439	0.615	9.671		
High dysfunction	1			1.000				
**Alcohol abuse**								
Yes	4.654	2.222	9.748	5.149	2.328	11.384	16.382	0.000^*^
No	1	–	–	1.000				
**Psychoactive substances consumption**								
Yes	0.458	0.307	0.684	0.613	0.389	0.966	4.442	0.035
No	1	–	–	1.000				

PRc, prevalence ratio—crude; PRa, prevalence ratio—adjusted; CI, confidence interval; Ll, lower limit; Lu, upper limit; X^2^, chi-squared.

^*^p statistically significant (*p* < 0.025).

In this context, we find education as a factor correlated with the Mental Health Index; however, statistical significance is insignificant among those who only completed Primary, Secondary, and Higher Technical education, showing an increase in PRs for those with Postgraduate degrees compared to crude measures and manifesting as a risk factor.

**Figure 2:**
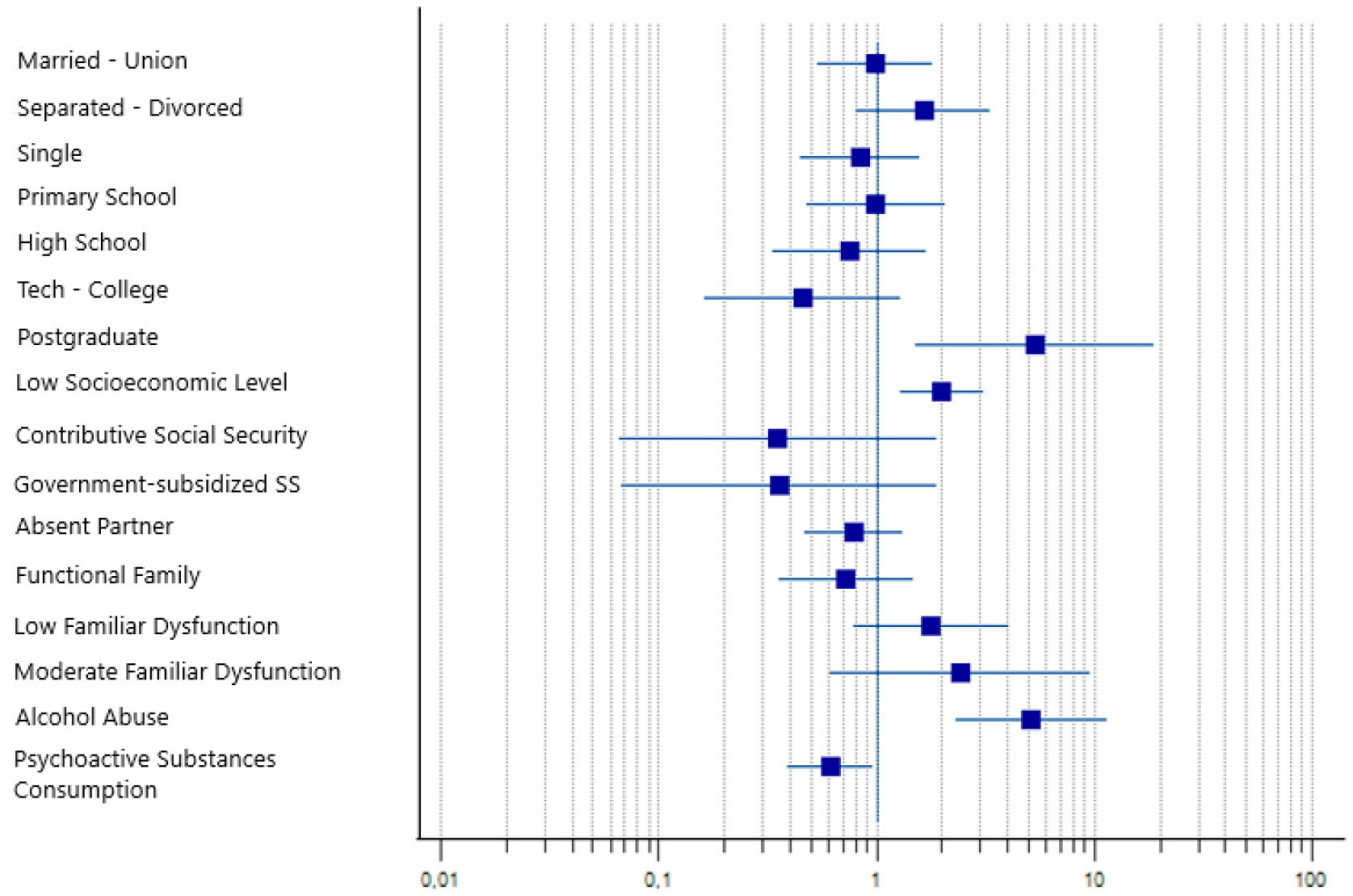
Factors associated with the mental health index in people over 60 years of age in Medellín, 2021, shown by a forest plot diagram. SS, social security.

The socioeconomic stratum showed a strong correlation even in adjusted measures without major variability compared to crude ones, maintaining statistical significance. We can infer from these values that the low stratum (1–2) is a risk factor (Figure 2).

Despite being statistically significant by *p*-value, the relationship of family APGAR shows that categories decrease association strength with a decrease in adjusted PRs compared to crude ones, compromising the correlation factor.

Finally, for alcohol abuse, an increase is observed in adjusted measures in those who consume alcohol, confirming it as a risk factor with adequate statistical significance.

## 4 Discussion

This study discusses several points that highlight sociodemographic, economic, and mental health inequities, highlighting the interest in making visible needs and requirements in public health of the population of people over 60 years in the city of Medellín and framed in the model of social determinants of health. The population profile managed in this study includes an older person mostly female (3:2), between 63 and 72 years old, with a stable marital union, education mainly up to primary school, where up to almost all have no income, or have an income of a legal monthly minimum wage, classified in low socioeconomic strata (1 and 2), with access to health insurance system in the subsidized regime, and very low benefit from the pension system. Similar to these data, in 2016, Arango described a “feminization of aging” for the population over 60 years in the city of Medellín (Cardona, 2016) given the longer life expectancy in women with an average of 6.8 years more than men according to DANE figures, however, there are no gender-differentiated studies to confirm differences in variable behavior.

In South America, an increase in the life expectancy of men and women has positioned Chile in the first place. Likewise, the consumption of alcohol exhibited notable significance in the exacerbation of mental health decline. Conversely, a data outlier arises, indicating diminished levels of mental health indices among individuals aged 60 and above holding postgraduate qualifications, devoid of prior precedent. While Chile presents life expectancy levels of both men and women to be in similar situations, in other countries on the continent, life expectancy levels can reach differences of >7 years. It is observed that in Chile there is a difference of 5 years in life expectancy at birth according to gender; a value lower than that presented by Venezuela (9.5 years difference) and at least 2 years lower than those of Uruguay, Brazil, and Colombia with 7.6, 7.5, and 7.3 years, respectively (Madero-Cabib et al., 2022).

The social determinants of the health model also include physical wellbeing conditions that include mental health. In this case, there is a population that reports a low frequency of physical illnesses, mostly with fully independent functionality, who in their habits present regular alcohol consumption in up to a third of the population, mainly in strata 1 and 2. These findings are relevant, as studies carried out in the region of the Americas will face a rapid process of population aging. This process is expected to accelerate by 2030 when it is estimated that 17% of the population with altered dependence will be over 60 years. It is important to clarify that this process of aging and loss of independence will occur in Latin America and the Caribbean in less than half the time it occurred in Europe (Cafagna et al., 2019). Considering this prevalence is important, it is estimated that the prevalence of dependence in older people could triple by 2050 (Aranco et al., 2018). The consumption of psychoactive substances was present in up to a third of the population, with the highest frequency being male consumers. In social relationships, we find that two-thirds of the studied population have a partner, with good family functionality in up to about half of the population, and with social support in all. The mental health of the evaluated population was characterized by a perception of medium/low quality of life in women, associated with the appearance of depressive symptoms of moderate intensity in the lower strata (1 and 2). Low levels on the resilient coping scale were predominant among men and women with low educational levels (primary and secondary), however, high self-esteem values were common among two-thirds of the population. The self-compassion scale showed a moderate range of values in all age groups regardless of sex, as well as percentages of mild and moderate hopelessness in ~80% of the population. In sync with the Colombian Policy on Human Aging and Old Age 2015–2024, in the line of action that aimed at protecting and guaranteeing the right to health, the policy proposes modifications to the health system and services that guarantee comprehensive care, better access for older adults to service provision according to their morbidity and mortality profiles, to guarantee a dignified life for them and their caregivers, and to foster aging with low prevalences of functional dependence and disability (Ministerio de Salud y Protección Social de Colombia, 2015). In this context, it is important to detect those habits and lifestyles that may lead to physical and mental complications in the future. In this way, it opens the possibility of characterizing a morbidity and mortality profile associated with mental health that can lead to new clinical knowledge in outpatient or hospital care settings for preventive and therapeutic applications.

The World Health Organization in 2020, which aimed to unite governments, civil society, international organizations, professionals, academic institutions, media, and the private sector through ~10 years of concerted action, catalytic, and collaborative action to improve the lives of older people, their families, and communities where they live, developed the Decade Plan for Healthy Aging 2020–2030 (WHO, Aging and Health Team, 2020). Within the paradigm of the Sustainable Development Goals, this project was conceptualized, where the health and wellbeing section seeks healthy aging aimed at older people contributing to society for longer periods, with opportunities for good health at all stages of life, universal health coverage, and integrated social and health systems, transformative and people-centered systems, rather than systems based solely on disease, are included. This includes an item exclusively dedicated to mental health that seeks to reduce suicide rates, as differentiated by age and sex. In this context, this research work allowed the development of a measurable indicator that facilitated the approach to a state of global and individual mental health in the population of people over 60 years. The indicator allowed the early detection of possible warning signs of psychiatric illness and the prevention of fatal outcomes from this etiology is applied to the population of the city of Medellín.

In this way, it ensures healthy comprehensive management both physical and mental, in addition to adequate social interaction and participation. In the development of the factorial analysis that allowed for establishing the main components of the mental health index, variables that are mostly part of the Self-Compassion Scale (SCS) stood out, showing the relevance of self-judgment, isolation, overidentification, self-kindness, humanity, and mindfulness in defining mental health. Currently, few studies examine the role of self-compassion in the context of social life, despite its potential in improving interpersonal relationship skills. It is determined that there are correlations between social security and the pleasure scale, with positive results in the factors of self-compassion, self-kindness, common humanity, and mindfulness. Conversely, self-judgment, isolation, and factors of excessive identification within self-compassion were identified negatively about social security. Additionally, the latter is positively correlated with awareness, self-kindness, and common humanity. It is also determined that isolation is correlated negatively. All this highlights the importance of self-compassion in social adjustment, highlighting the importance of these findings concerning existing literature (Akin and Akin, 2015).

In this regard, in the city of Medellín in 2021, a general state of mental health was found to be measured by the Mental Health Index among people over 60 years with a negative correlation reaching 55% of the population. Challenged with this, it was possible to relate statistical significance to three risk factors that may predispose to the outcome. The first finding was alcohol the association between alcohol consumption and negative outcomes in the mental health index, consistent with official national figures. Estimates of the prevalence of alcohol-related problems in people over 60 years are found to be between 1 and 6% of the general population in Colombia according to the National Mental Health Survey 2015 (Ministerio de Salud y Protección social, Colombia, 2015), 7–22% of patients in general hospitalization, and 28–44% of patients hospitalized for psychiatric pathology. This finding highlights a point of intervention with significant importance in the prevention and management of mental pathology in the population over 60 years, initially focusing on the city of Medellín, with the intention to apply in the future at the national level or other populations within the same age range (De La Espriella Guerrero et al., 2016).

It is striking evidence that postgraduate schooling is an associated factor with negative mental health indices when antecedents do not favor outcomes. In review literature for some Latin American countries such as Mexico, no association was found between degree schooling and with the appearance of mental symptoms such as depressive disorders (Mundo-Rosas et al., 2011). In Colombia, in the year 2012, Segura and Cardona concluded a protective relationship at a higher level of schooling for the development of depressive symptoms (Segura Cardona et al., 2015). In a random qualitative survey of people in the same age range, where potential causes of this finding are investigated, it emerges as an assumption of a “loss of value at a professional, work and academic level as age progresses.” This result opens the door to future research and intervention points in specific populations among adults over 60 years.

In 2015, a study was conducted on mental health in older people treated in the public hospital network in the city of Medellín, where they found that more than 85% of patients with poor mental health belong to low socioeconomic strata (Agudelo-Suárez et al., 2015). In contrast, this research does not limit the population to hospitalized patients from the public network, as the sample was randomized in different communes of the city, reporting the frequency of low socioeconomic stratum in 66.5% of the population, with a strong correlation with a low mental health index. In a systematic review of the literature, it was found that in Latin America, the authors associate the deterioration of mental health with low socioeconomic strata and alcohol consumption simultaneously (Ortiz-Hernández et al., 2007). These data confirm and highlight a social problem in the city that has not received any type of public health intervention.

The limitations in this study may be associated with obtaining data from secondary sources. For this reason, the information could have been affected by registration and/or data collection. However, methodological rigor was ensured through bias control and database management to guarantee the validity and reliability of the results.

## 5 Conclusion

As a first conclusion, we can affirm that, with the model designed for this study, it was possible to develop the Mental Health Index and thus validate that the initially proposed null hypothesis is fulfilled. This hypothesis aimed to find a relationship between sociodemographic factors and physical and lifestyle habits of people over 60 years with the state of mental health calculated by this Index. This study established the most important domains that may be involved in the development of positive or negative aspects in the mental health of the currently studied population. Among these, its main objective is to develop actions that intervene in improving health and therefore quality of life in this group of people.

It was possible to establish a sociodemographic profile that reflects economic inequities and social gaps that, together with unhealthy lifestyles and habits, contextualize an environment that can trigger the physical and mental deterioration of older people in the Medellín area. The study also established that having inadequate housing for older adults and living in low social strata is a negative factor in guaranteeing adequate mental health in older adults. In this regard, it was necessary to develop a measurement model based on the evaluation of multiple relevant aspects contributing to psychological wellbeing. The final product of this effort was an index that allowed quantitative estimation of mental health status.

The Mental Health Index allowed exposing low levels of mental wellbeing in older people from the city of Medellín in the year 2021. In addition, it was possible to observe the association between sociodemographic factors and physical and lifestyle habits that can trigger this finding.

It is not surprising that, in an environment characterized by social inequities, the association between a negative Mental Health Index and having a low socioeconomic stratum is a determining factor. Similarly, alcohol consumption showed special relevance for the deterioration of mental health. On the other hand, a data point without antecedents emerges when exposing low values of the Mental Health Index in people over 60 years with a postgraduate level of education. These factors generate a need for intervention and a possibility of future research for this population.

## Data availability statement

The original contributions presented in the study are included in the article/Supplementary material, further inquiries can be directed to the corresponding author.

## Ethics statement

The studies involving humans were approved by Ethics Committee of CES University, classified as minimal risk (Act 134 of May 23, 2019). The studies were conducted in accordance with the local legislation and institutional requirements. Written informed consent for participation was provided by the participants.

## Author contributions

SO-N: Conceptualization, Formal analysis, Writing—original draft, Writing—review & editing, Data curation, Methodology. LB-G: Conceptualization, Data curation, Formal analysis, Writing—original draft, Writing—review & editing. DC-A: Conceptualization, Formal analysis, Writing—original draft, Writing—review & editing, Investigation, Project administration. AnS-C: Data curation, Formal analysis, Methodology, Supervision, Validation, Writing—review & editing. AlS-C: Conceptualization, Formal analysis, Investigation, Supervision, Writing—original draft. CR-M: Investigation, Writing—review & editing.
