# Supplementary material for: Mental health index of the elderly population in Medellín (Colombia)−2021: a factorial analysis
Source: Front Psychol. 2024 Jun 26;15:1336562. doi: 10.3389/fpsyg.2024.1336562 (PMC11233805; doi:10.3389/fpsyg.2024.1336562)
Supplement: Supplementary file 1 [file Table_1.docx]

**SUPPLEMENTARY MATERIAL**

Description of the variables used for data analysis and construction of the Mental Health Index in the Elderly.

**Table 1. Sociodemographic and Family Variables**

| **VARIABLE** | **OPERATIONAL DEFINITION** | **TYPE** | **UNITS** | **CATEGORIES** | **MEASUREMENT LEVEL** |
| --- | --- | --- | --- | --- | --- |
| Age | Number of years completed | Quantitative | Years completed |  | Categorical |
| Sex | Biological condition at birth | Qualitative Dichotomic |  | 1. Masculine 2. Feminine | Nominal |
| Marital Status | Actual marital status | Qualitative Polytomic |  | 1. Single 2. Married - Union 3. Separated- Divorced 4. Widow | Nominal |
| Education | The highest level of schooling attained | Qualitative Polytomic |  | 1. Primary school 2. High school 3. Tech – University 4. Postgraduate 5. None | Nominal |
| Socioeconomic stratum | Classification by neighborhood | Qualitative Dichotomic |  | Low (1 -2)  Medium (3 – 4) | Ordinal |
| Social Security in Health | Affiliated | Qualitative Dichotomic |  | 1. Yes 2. No | Nominal |
| Social Security – Type | Type of affiliation in Social Security in Health | Qualitative Polytomic |  | 1. Contributive 2. Subsided 3. None | Nominal |
| Pension program | Affiliated | Qualitative Dichotomic |  | 1. Yes 2. No | Nominal |

**Corregimiento*

**Table 2. Clinical and lifestyle variables**

| **VARIABLE** | **OPERATIONAL DEFINITION** | **TYPE** | **UNITS** | **CATEGORIES** | **MEASUREMENT LEVEL** |
| --- | --- | --- | --- | --- | --- |
| Illness | Confirmed Diagnosis | Qualitative Dichotomic |  | - 1. Yes   2. No | Nominal |
| Barthel | Barthel Functional Scale | Qualitative Polytomic |  | Independiente  Low Dependency  Moderated Dependency  Severe Dependency  Total Dependency | Ordinal |
| Barthel Record | Dichotomic Barthel Functional Scale | Qualitative Dichotomic |  | Independent  Dependency | Ordinal |
| Alcohol Abuse | Story of Alcohol Abuse | Qualitative Dichotomic |  | - - 1. Yes     2. No | Nominal |
| Psychoactive substances consumption | Any substance? | Qualitative Dichotomic |  | - - - 1. Yes       2. No | Nominal |

**Table 3. Interpersonal Relationship Variables**

| **VARIABLE** | **OPERATIONAL DEFINITION** | **TYPE** | **UNITS** | **CATEGORIES** | **MEASUREMENT LEVEL** |
| --- | --- | --- | --- | --- | --- |
| Absent Partner | Is your partner absent? | Qualitative Dichotomic |  | - 1. Yes   2. No | Nominal |
| Familiar APGAR | Familiar APGAR Scale | Qualitative Polytomic |  | Functional  Dysfunction - Low  Dysfunction - Moderated  Dysfunction - Severe | Ordinal |
| Deficient Social Support | Report | Qualitative Dichotomic |  | - - 1. Yes     2. No | Nominal |

**Table 4. Mental Health Scale Measurement Variables**

| **VARIABLE** | **OPERATIONAL DEFINITION** | **TYPE** | **UNITS** | **CATEGORIES** | **MEASUREMENT LEVEL** |
| --- | --- | --- | --- | --- | --- |
| Quality of Life | WHOQOL-OLD Scale | Qualitative Polytomic |  | High  Medium  Low | Ordinal |
| Depression Value | Center for Epidemiological Studies Depression Scale – CES D | Quantitative | None |  | Categorical |
| Depression | Center for Epidemiological Studies Depression Scale – CES D | Qualitative Polytomic |  | None  Low  Moderated  Severe | Ordinal |
| Resilient Coping Value | Brief Resilient Coping Scale | Quantitative | None |  | Categorical |
| Resilient Coping | Brief Resilient Coping Scale |  |  | Low  Moderated  High | Ordinal |
| Self-Esteem Value | Rosenberg Self-Esteem Scale | Quantitative | None |  | Categorical |
| Self -Esteem | Rosenberg Self-Esteem Scale | Qualitative Polytomic |  | Low  Medium  High | Ordinal |
| Hopelessness value | Beck Hopelessness Scale | Quantitative | None |  | Categorical |
| Hopelessness | Beck Hopelessness Scale | Qualitative Polytomic |  | Asymptomatic  Low  Moderated  Severe | Ordinal |
| Self-compassion Value | Self-compassion Scale (SCS) | Quantitative | None |  | Categorical |
| Self-compassion | Self-compassion Scale (SCS) | Qualitative Polytomic |  | Low  Moderated  High | Ordinal |
| Self-care | SCS Self-Care Domain | Quantitative | None |  | Categorical |
| Isolation | SCS Isolation Domain | Quantitative | None |  | Categorical |
| Overidentification | SCS Overidentification Domain | Quantitative | None |  | Categorical |
| Self - Kindness | SCS Self - Kindness Domain | Quantitative | None |  | Categorical |
| Humankind | SCS Humankind Domain | Quantitative | None |  | Categorical |
| Mindfulness | SCS Mindfulness Domain | Quantitative | None |  | Categorical |

**Table 5. Variables obtained from the Mental Health Index in the Elderly in Medellín, 2021**

| **VARIABLE** | **OPERATIONAL DEFINITION** | **TYPE** | **UNITS** | **CATEGORIES** | **MEASUREMENT LEVEL** |
| --- | --- | --- | --- | --- | --- |
| Individual | Component 1 values obtained from factor analysis | Quantitative | None |  | Continuous |
| Existential | Component 2 values obtained from factor analysis | Quantitative | None |  | Continuous |
| Mental Health Index Summary | Component 1 and 2 values obtained from factor analysis summary | Quantitative | None |  | Continuous |
| Mental Health Index | Classification | Qualitative Dichotomic |  | Positive  Negative | Nominal |
